# A non-destructive testing method for early detection of ginseng root diseases using machine learning technologies based on leaf hyperspectral reflectance

**DOI:** 10.3389/fpls.2022.1031030

**Published:** 2022-11-17

**Authors:** Guiping Zhao, Yifei Pei, Ruoqi Yang, Li Xiang, Zihan Fang, Ye Wang, Dou Yin, Jie Wu, Dan Gao, Dade Yu, Xiwen Li

**Affiliations:** ^1^ Institute of Chinese Materia Medica, China Academy of Chinese Medical Sciences, Beijing, China; ^2^ College of Traditional Chinese Medicine, Yunnan University of Chinese Medicine, Kunming, China; ^3^ School of Pharmacy, Shandong University of Traditional Chinese Medicine, Jinan, China; ^4^ TCM Department, China National Center for Biotechnology Development, Beijing, China; ^5^ School of Basic Medical Sciences, Anhui Medical University, Hefei, Anhui, China

**Keywords:** ginseng root diseases, hyperspectral reflectance, vegetation indices, extreme random tree, specific band

## Abstract

Ginseng is an important medicinal plant benefiting human health for thousands of years. Root disease is the main cause of ginseng yield loss. It is difficult to detect ginseng root disease by manual observation on the changes of leaves, as it takes a long time until symptoms appear on leaves after the infection on roots. In order to detect root diseases at early stages and limit their further spread, an efficient and non-destructive testing (NDT) method is urgently needed. Hyperspectral remote sensing technology was performed in this study to discern whether ginseng roots were diseased. Hyperspectral reflectance of leaves at 325-1,075 nm were collected from the ginsengs with no symptoms on leaves at visual. These spectra were divided into healthy and diseased groups according to the symptoms on roots after harvest. The hyperspectral data were used to construct machine learning classification models including random forest, extreme random tree (ET), adaptive boosting and gradient boosting decision tree respectively to identify diseased ginsengs, while calculating the vegetation indices and analyzing the region of specific spectral bands. The precision rates of the ET model preprocessed by savitzky golay method for the identification of healthy and diseased ginsengs reached 99% and 98%, respectively. Combined with the preliminary analysis of band importance, vegetation indices and physiological characteristics, 690-726 nm was screened out as a specific band for early detection of ginseng root diseases. Therefore, underground root diseases can be effectively detected at an early stage by leaf hyperspectral reflectance. The NDT method for early detection of ginsengs root diseases is proposed in this study. The method is helpful in the prevention and control of root diseases of ginsengs to prevent the reduction of ginseng yield.

## 1 Introduction

**Figure 1:**
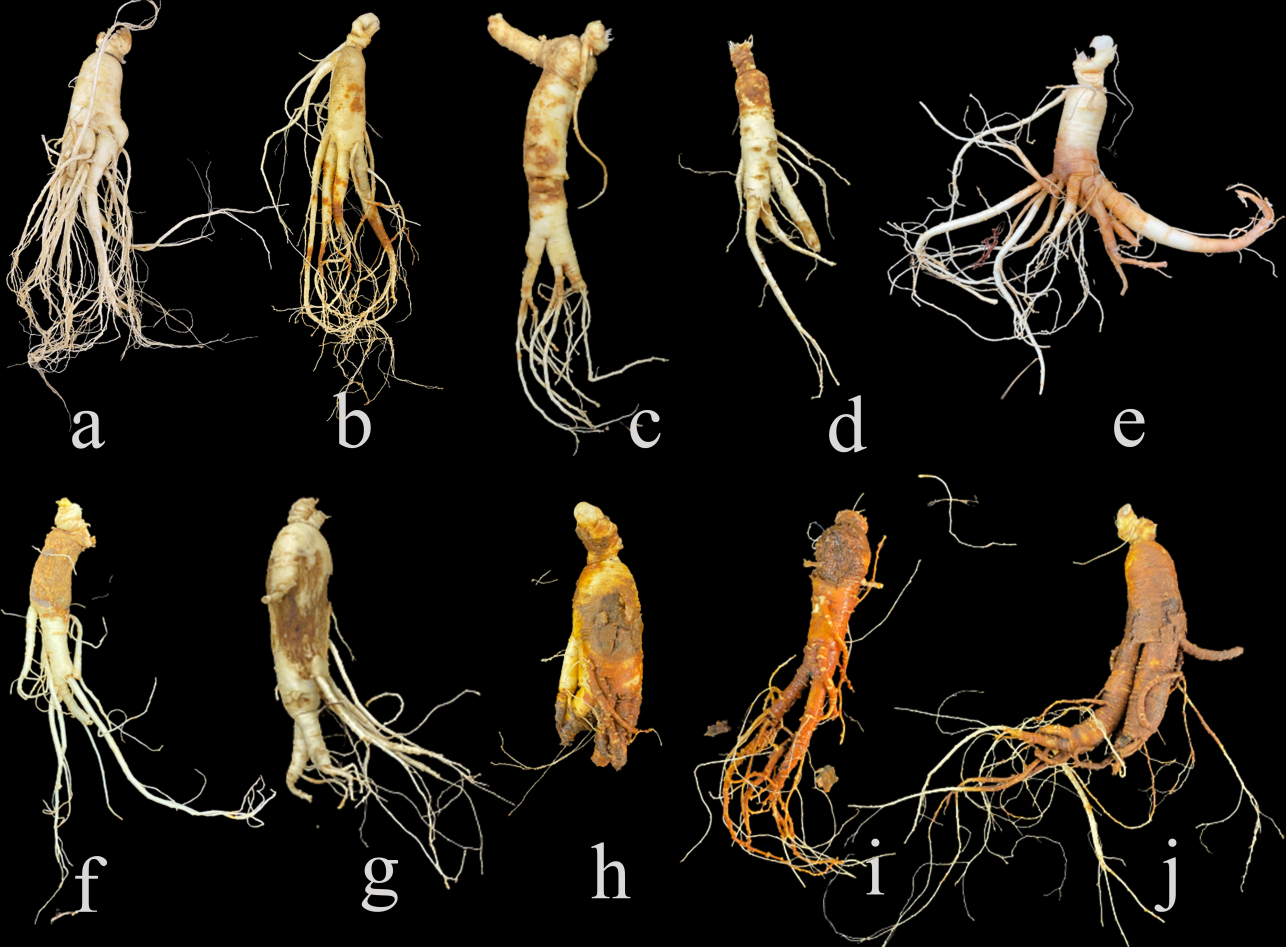
Symptoms of diseased ginseng roots collected in the field. **(A)** is healthy root, **(B–J)** are various diseased roots.

Ginseng (*Panax ginseng* Mey) is one of the precious traditional herbs. The roots of ginseng are widely used as important medicinal materials for curing hypertension, stress, and neurological disorders (Ratan et al., 2021). Nowadays, wild ginsengs are endangered and cultivated ginsengs are used as main resources of ginseng products (Xu et al., 2016). The harvest rotation of cultivated ginseng is usually about 4 to 6 years (Xiao et al., 2016). Ginsengs cultivated in the same soil for a long-growth period are susceptible to root diseases (Fang et al., 2022), such as root rot, rusty root rot, red-skin, soft rot and so on (Liang et al., 2017; Lu et al., 2020). Root disease in ginseng is caused by a variety of factors, including biological factors such as pathogen infection, and abiotic factors such as soil temperature and moisture (Shang et al., 1996; Zhou et al., 2017), leading to diverse symptoms (**Figure 1**). The incidence rate of ginseng roots can be as high as 80% (Wang et al., 2014), which seriously reduces the production of ginseng and costs huge economic losses. So far, the commonly test method of ginseng root diseases in the field mainly relies on experiences of farmers by visually observing the symptoms of aerial parts (Farh et al., 2018), which is extremely difficult to generate and has poor accuracy, especially at the early stages of root diseases when there are no macroscopic symptoms on leaves can be observed (Lu et al., 2020). When there are visible lesions on the aboveground plant, the root has rotted and it was too late for treatment (Guan et al., 2014). Unnecessarily damages or losses are caused artificially if ginseng roots were dug out for visual detection, though it is more precise by visualization on the underground part. Thus, it is importance to detect the root diseases at their early stages, as that treatments could be taken in time.

Root diseases were often accompanied with a decrease in chlorophyll content of leaves (Fujimoto et al., 2021; Jia et al., 2021), which indicated the reduction of plant photosynthesis and external stresses (Monteoliva et al., 2021). In addition, the contents of antioxidant enzymes in leaves are also affected by root diseases (Harrach et al., 2013). The increase of enzyme activity improves the tolerance of plants to stress environments (Adavi et al., 2020; Deng et al., 2020). For example, in wheat, the antioxidant enzyme activities increased in resistance to the Bipolaris sorokiniana-common root rot (Qalavand et al., 2022). Chlorophyll content and antioxidant enzyme activity of leaves are physiological characters responding to nutrient deficiency, abiotic stresses and biotic stresses (Panda and Sarkar, 2013), the changes of which could be considered as potential indicators of root diseases of plants.

Nowadays, hyperspectral remote sensing technology has been widely used for rapid and non-destructive testing (NDT) of plant diseases (Lowe et al., 2017; Mahlein et al., 2018; Chen et al., 2022), such as wheat powdery mildew (Feng et al., 2022), fusarium head blight (Zhang et al., 2020) and decayed citrus (Zhang et al., 2021). The effective information on plant growth status is archived by screening and processing the complex and redundant hyperspectral data (Yang et al., 2020). Hyperspectral vegetation indices (VIs) were widely deployed to estimate plant biophysical and biochemical traits (Koh et al., 2022). The downy mildew severity stages in watermelon are significantly correlated with the chlorophyll green, photochemical reflectance index and normalized phaeophytinization index (Abdulridha et al., 2022). Improved accuracy of hyperspectral data processing due to advances of machine learning (ML) enabling further development of non-invasive high-throughput plant phenotyping (Mahlein et al., 2019; Arya et al., 2022). ML algorithms such as random forest (RF), support vector machine and convolutional neural networks, have improved the accuracy of hyperspectral data processing to obtain the spectral characteristics of plants during the growth period, and to finalize the early detection of plant diseases (Ghosh et al., 2022), such as the Fusarium head blight disease severity of wheat (Żelazny et al., 2021). Combined with artificial intelligence algorithms and continuous rich hyperspectral reflectance data, models could be constructed to detect and identify plant diseases (Singh et al., 2018; Singh et al., 2021). The application of ML technologies based on hyperspectral reflectance enables precise diagnosis of plant root diseases at an early stage.

To prevent the massive loss of ginseng yield caused by severe root diseases, this study proposed an NDT method using the hyperspectral remote sensing technology to detect root diseases of ginsengs at their early stages. Based on the previous field investigation, ginsengs with healthy leaves in the field of high incidence of root diseases were used. The hyperspectral reflectance data of ginseng leaves were obtained non-destructively to construct a detection model by ML. Taking the constructed model as a reference, a hyperspectral inversion model of ginseng root disease was established. We finally found that hyperspectral remote sensing technology could achieve early accurate detection of ginseng root diseases, which greatly reduced the loss of production and avoided the use of excessive pesticides.

## 2 Materials and methods

### 2.1 Experiment setup and measurements in the field

The experimental site was located at the experimental base of the Chinese Academy of Traditional Chinese Medicine in Jingyu County, Jilin Province, China (126.8°E, 42.39°N), and the ginsengs were cultivated continuously for 3-5 years. The cultivated soils were all farmland soils where ginseng had not been grown before, and the ginseng was cultivated in shade by covering with blue and yellow shade nets. The field management, including watering and fertilizing, was the same as the cultivated ginsengs in farmland as has been described by Shen et al. (2017). The measurements and harvest were conducted in 17 to 20 August 2021, a period when ginseng grows in the red-fruited stage and the average local temperature was around 15°C to 25°C. A total of 217 ginsengs with no diseased symptoms on leaves were chosen in the field for the measurements. The leaf chlorophyll contents were measured by soil plant analysis development chlorophyll meter (SPAD-502 Plus, KONICA MINOLTA, Japan) with the middle leaf of a palmately compound leaf. Three middle leaves of each ginseng were measured and each leaf was measured three times. The hyperspectral reflectance of the middle and upper parts of the largest leaf of these ginsengs were collected using a FieldSpec HandHeld 2 Spectroradiometer (HH2, Analytical Spectral Devices, Colorado, US). The wavelength of HH2 is 325-1,075 nm, and the sampling interval of HH2 is 1.4 nm. It is used in combination with a plant probe, an optical fiber and a leaf clip to avoid the disturbances of ambient light. The spectral reflectance was measured 10 times per leaf using white plate calibration in prior to measurement.

### 2.2 Sample collection and antioxidant enzyme measurements

**Figure 2:**
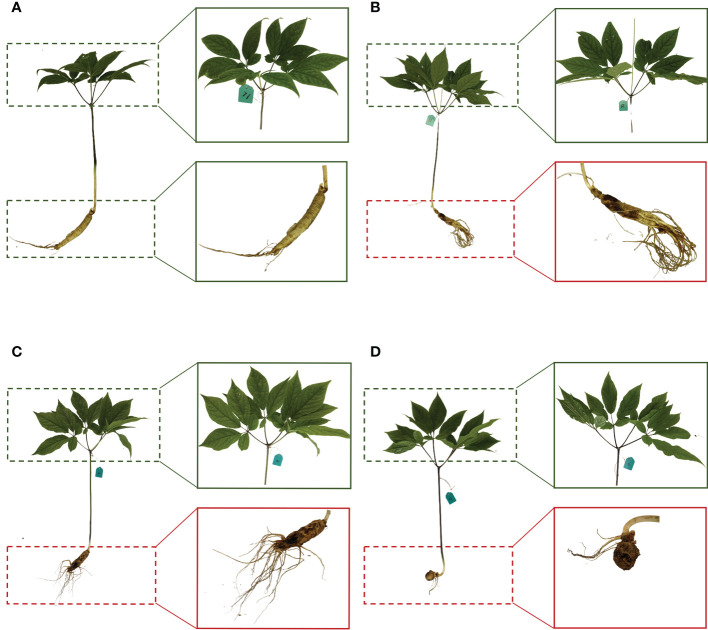
Whole plant, leaves, and root of representative healthy ginseng **(A)** and asymptomatic diseased ginsengs **(B–D)**. The dashed green box represents the healthy part, the dashed red box represents the diseased part.

After the measurement of spectral reflectance, the 217 ginseng plants were harvested. Roots with stem and leaves were dug out. They were washed with running water and thereafter dried by tissue paper. Pictures of each plant were taken, and healthy and diseased ginseng roots were separated by visual observation on whether there was disease spot on the surface or rot part. The leaves were removed from each stem, shortly frozen in the liquid nitrogen and then stored at -80°C. Based on the visually assessed symptoms of ginseng roots, 57 samples were healthy, and 160 samples were diseased (**Figure 2**).

The Frozen leaves of randomly selected 15 healthy and 15 diseased ginsengs were used for antioxidant enzyme activity measurement. Three leaves of each ginseng (~ 0.2 g) were weighed for these measurements. The superoxide dismutase (SOD) enzyme activity of ginseng leaves was determined by nitro blue tetrazolium photoreduction method, peroxidase (POD) enzyme activity was determined by guaiacol method, and catalase (CAT) enzymes activity was determined by ultraviolet spectrophotometry, as described previously by Li (2000).

### 2.3 The calculation of spectral vegetation indices

In this study, 8 vegetation indices that related to plant stresses (Ayanlade, 2017; Xue and Su, 2017; Velichkova et al., 2019) were calculated (details see the supplementary, **Table S1**). These vegetation indices are mainly types of narrowband greenness and leaf pigments. The red and near-infrared region (red edge) between 690-740 nm was used to calculate the narrowband greenness.

### 2.4 Preprocessing of hyperspectral reflectance

The raw data of hyperspectral reflectance of ginseng leaves were exported by the spectral data processing software View Spec Pro (Analytical Spectral Devices, Colorado, US) from the spectrometer. Machine noises at the beginning and the end of a band were removed according to the fluctuation range of the hyperspectral reflectance curve, and the spectral data with less machine noises were selected for subsequent processing. In this study, 10 preprocessing schemes including first derivative (1D), second derivative (2D), standard normal variate (SNV), multiple scattering correction (MSC), savitzky-golay (SG), 1D-SG, 2D-SG, SNV-SG, and MSC-SG were used, which were completed by the software SIMCA_P+ 13.0 (Umetrics, Umea, Sweden). SNV is used to correct for light scattering differences. MSC effectively eliminates spectral differences due to different scattering levels. Derivative processing effectively improves sensitivity and resolution. SG preprocessing smoothest high-frequency noise and effectively improves the signal-to-noise ratio. The fluctuation range and smoothness of spectral reflectance were compared according to the image, and the accuracy was compared according to the preliminary modeling results. The best preprocessing method was selected and then further modeling was performed.

### 2.5 Construction of detection models and evaluation indices

Based on the Python 3.7.0 open-source ML toolkit Scikit-Learn (Pedregosa et al., 2011), models were built using 4 algorithms (RF; ET, extremely randomized trees; ADA, adaptive boosting; GBDT, gradient boosting decision tree) with the hyperspectral dataset of ginseng leaves after optimal preprocessing. The basic unit of RF is a single decision tree, and its main idea is ensemble learning (EL). Every decision tree in a RF is a classifier. For an input sample, the RF algorithm assigns the class with the most votes as the final output by performing an equal vote on the predictions of all decision trees (Breiman, 2001). The ET algorithm is very similar to the RF algorithm. The difference between the two is that the training set of each decision tree in the RF model is obtained by random sampling, while each decision tree in the ET model uses the original training set (Geurts et al., 2006). Therefore, the variance of the ET algorithm is lower than that of RF to some extent, but its bias is relatively high. Furthermore, unlike RF which selects the optimal eigenvalue split point, ET usually randomly selects an eigenvalue split point. ADA is an ensemble algorithm based on the boosting strategy. Its core idea is to use the training set to train multiple weak classifiers. During this process, the weight of the sample and the weight of the classifier are constantly changing. Finally, a strong classifier is formed. The “adaptation” of the ADA algorithm is reflected in the fact that the weight of each sample is determined by the accuracy of the previous prediction (Freund and Schapire, 1997; Bauer and Kohavi, 1999). GBDT is also an EL based on boosting strategy, but it is different from the ADA algorithm. For the GBDT algorithm, it does not have the concept of sample weight, but adopts the concept of “residual error”. In detail, the GBDT algorithm is fitted for the negative gradient of the current model, and in this process, the error value of the weak learner is getting smaller and smaller (Friedman, 2001). Since 10 measurements were recorded for one plant, there were a total of 2 170 groups of data for all plants. These data were divided randomly into the training set and test set with the ratio of 3:1. The healthy ginseng was represented by 0, and the diseased ginseng was represented by 1. The training set was used for model establishment and optimization, and the test set was used to test the hyperspectral reflectance data collected from ginseng leaves. Then Grid Search and Learning Curve were combined to adjust the hyperparameters of the model, including n_estimators, max-depth, and subsample, so as to achieve the purpose of optimizing the model (Isa et al., 2019). The optimal hyperparameter combination for model optimization was set as follows: n_estimators = 100, max_depth = 17 for RF model; n_estimators = 50, max_depth = 19 for ET model; n_estimators = 240 for the ADA model; and n_estimators = 270, subsample = 0.7 for the GBDT model.

Finally, the importance of all hyperspectral bands was sorted, and the characteristic bands that played important roles in the early detection of ginseng root diseases were screened out. The performance of each model was compared by calculating different indicators, including accuracy, precision, recall, f1-score, area under the curve (AUC) and Matthew’s correlation coefficient (MCC). Among the indicators, precision represents the proportion of truly diseased samples to the samples predicted to be diseased by the model; recall represents the proportion of all diseased samples that the model predicted correctly; and f1-score is the balance coefficient between precision and recall. By drawing receiver operating characteristic curve (ROC) and confusion matrix chart, the performance of the model itself and the overall prediction effect were objectively evaluated. The specific calculation formula are as follows:


(1) Accuracy=(TP+TN)/(TP+FP+TN+FN)

(2) Precision=TP/(TP+FP)

(3) Recall=TP/(TP+FN)

(4) F1−score=2×Precision×Recall/(Precision+Recall)

Where TP (true positive) indicates the number of diseased ginsengs correctly predicted, TN (true negative) indicates the number of healthy ginsengs correctly predicted, FP (false positive) indicates the number of healthy ginsengs wrongly predicted, FN (false negative) indicates the number of diseased ginsengs wrongly predicted.

### 2.6 Model validation

Besides 217 ginseng plants, hyperspectral reflectance of leaves of randomly selected another 12 ginsengs with healthy leaves growing were also collected using HH2 in the same way. The roots of these 12 ginsengs were harvested for the classification of healthy or diseased ginsengs. The selected model was applied to predict these randomly selected 12 ginsengs. Ten groups of hyperspectral reflectance data were collected from each plant. The average ten groups of data of each plant were calculated and was used for the prediction. The predicted results of the model were compared to the classification after harvest and the prediction accuracy was calculated.

### 2.7 Statistical analysis

Statistical analysis in this study was conducted using SPSS 20.0 Software (IBM Corp., Armonk, NY, USA). SPAD values, antioxidant enzyme activities, and vegetative indices of ginseng leaves from both healthy and diseased groups were compared for differences using student T-tests.

## 3 Results

### 3.1 The chlorophyll content and the activity of antioxidant enzymes

**Figure 3:**
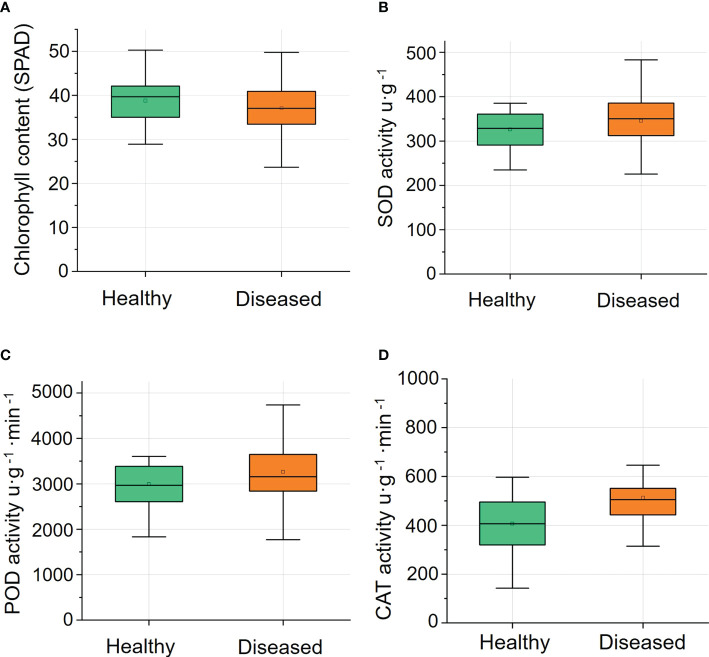
The chlorophyll content (n=57) and the activity of antioxidant enzymes (n=15) of leaves of healthy and diseased ginsengs. **(A)** Changes of relative chlorophyll content in healthy and diseased ginseng. **(B)** Changes of SOD activity in healthy and diseased ginseng. **(C)** Changes of POD activity in healthy and diseased ginseng. **(D)** Changes of CAT activity in healthy and diseased ginseng. SPAD, soil plant analysis development; SOD, superoxide dismutase; POD, peroxidase; CAT, catalase.

The chlorophyll contents of leaves of healthy ginsengs were slightly higher than those of leaves of diseased ginsengs, but this difference was not significant (**Figure 3A**). The absence of lesions on leaves inhibits us to recognize root diseases at their early stages. Similarly, the antioxidant enzyme activities of SOD, POD and CAT in leaves of diseased ginsengs were also slightly higher than those in leaves of healthy ginsengs (**Figures 3B–D**), but not significantly (n=15, P>0.05). Thus, the activities of antioxidant enzymes were in accordance with the macroscopic symptoms of leaves, and cannot be the indicator of root diseases.

### 3.2 Hyperspectral vegetation indices

**Figure 4:**
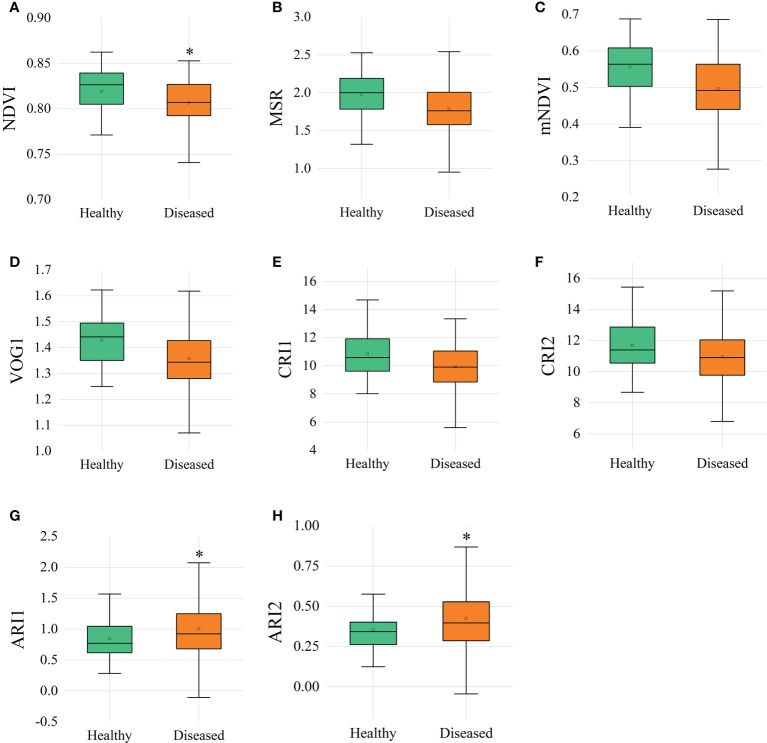
Vegetation indices of healthy and diseased ginsengs. n=57, * indicates the significant difference between healthy and diseased (P< 0.05). **(A)** NDVI, red edge normalized difference vegetation index; **(B)** MSR, modified red edge simple ratio; **(C)** mNDVI, modified NDVI; **(D)** VOG, vogelmann red edge index; **(E, F)** CRI, carotenoid reflectance index; **(G, H)** ARI, anthocyanin reflectance index.

VIs related to vegetation vitality, anthocyanin and carotenoid content were calculated with hyperspectral reflectance. The narrowband greenness values, such as red edge normalized difference vegetation index (NDVI), modified red edge simple ratio (MSR), modified NDVI (mNDVI), and vogelmann red edge index1 (VOG1), of diseased ginseng leaves were lower than those of healthy ginsengs (**Figure 4**). Especially the value of NDVI, which was significantly lower (P<0.05) in the diseased ginsengs than in the healthy ginsengs, indicating that at the position of the red edge (690-740 nm), ginsengs with diseased roots could be identified by the NDVI value. In addition, the leaf pigments of diseased ginseng leaves, represented by anthocyanin reflectance index 1 (ARI1) and ARI2, showed significant higher values (P<0.05) than those of healthy ginseng leaves (**Figure 4**), indicating that the content of anthocyanins in diseased ginseng leaves had been increased, and senescence symptoms would be expected to appear in the next stage of the diseases. However, the VIs related to carotenoids, such as carotenoid reflectance index 1 (CRI1) and CRI2 values of diseased ginsengs, were slightly lower, but not significantly changed, than those of the healthy ginsengs (**Figure 4**).

### 3.3 Hyperspectral reflectance

#### 3.3.1 Pre-processing of hyperspectral reflectance data

**Figure 5:**
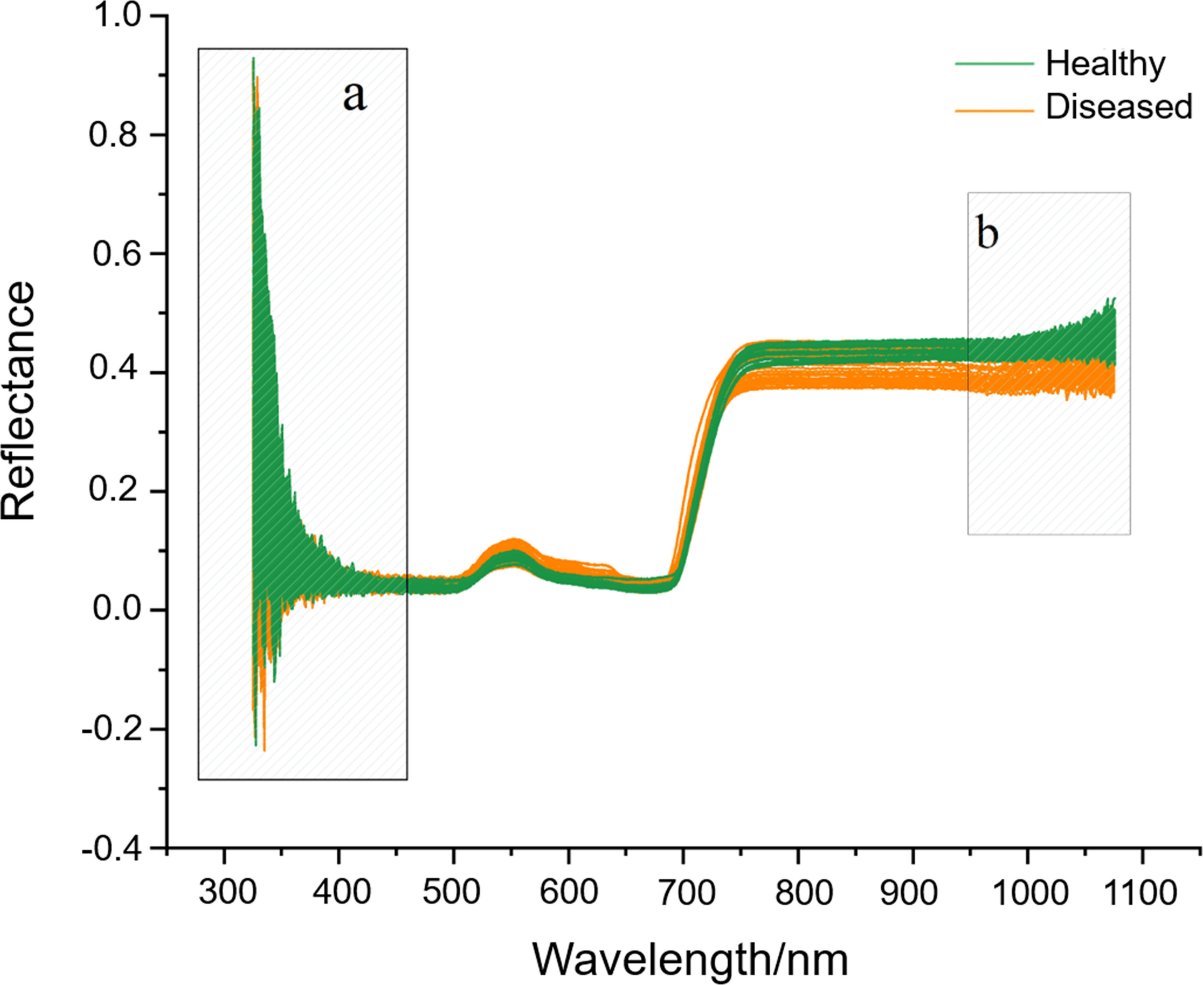
The original hyperspectral reflectance of ginseng leaves **(A, B)** are the band ranges with machine noises and are visually selected to be removed.

Hyperspectral bands and a large amount of reflectance data were obtained from ginseng leaves. We selected hyperspectral data with less noises in the region of 460-950 nm by removing the noises at both ends of hyperspectral bands (**Figure 5**). Among the 10 pre-processing methods (single methods of SNV, MSC, derivative and SG and their different combinations), the SG retained the trend of the original spectral curve, and the reflectivity range was concentrated in 0-0.5 with a clear curve outline (**Figure S1**).

The accuracy rates of RF, ADA, GBDT, and ET under the raw dataset conditions were 94.81%, 80.08%, 84.65%, and 97.13%, respectively (**Table 1**). Among the 10 pre-processing methods, after SG pre-processing, the models of RF, GBDT, and ET achieved the highest scores, which were 95.76%, 87.29%, and 97.97%, respectively. After SG pre-processing, the ADA model’s accuracy was 79.37%, which was a bit lower than the raw data and the data pre-processed after SNV-SG. Thus, based on the accuracy of the models and the changes of reflectivity curve, the method of SG for data pre-processing was taken as the best pre-processing method in this study.

**Table 1:** Accuracy rates of 10 preprocessing methods based on the 4 algorithms.

Accuracy (%)	Raw	1D	2D	SNV	MSC	SG	1D-SG	2D-SG	SNV-SG	MSC-SG
**RF**	94.81	86.56	78.64	87.66	86.92	95.76	91.97	92.45	87.58	79.23
**ADA**	80.08	77.35	72.38	78.45	77.72	79.37	79.19	78.82	81.49	72.46
**GBDT**	84.65	83.24	78.27	82.69	82.87	87.29	86.56	86.74	86.00	75.62
**ET**	97.13	89.32	78.27	91.53	91.16	97.97	96.69	96.13	92.10	80.14

RF, Random forest; ET, Extremely randomized trees; ADA, Adaptive boosting; GBDT, Gradient boosting decision tree; RAW, Raw dataset; 1D, first derivative; 2D, Second derivative; SNV, Standard normal variate; MSC, Multiple scattering correction; SG, Savitzky golay; 1D-SG, first derivative- savitzky golay; 2D-SG, Second derivative – savitzky golay; SNV-SG, Standard normal variate – savitzky golay; MSC-SG, Multiple scattering correction- savitzky golay.

#### 3.3.2 Model optimization and evaluation

**Figure 6:**
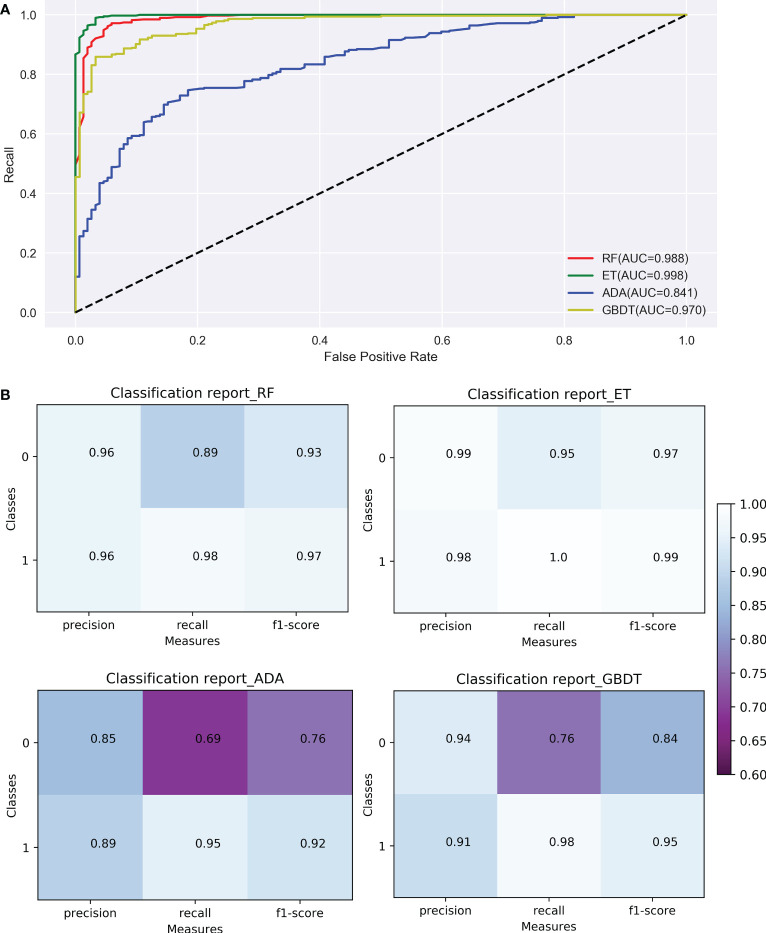
Comparison of ROC curves **(A)** and confusion matrix **(B)** of the 4 models (RF, ET, ADA, GBDT). RF, Random Forest; ET, Extremely randomized trees; ADA, Adaptive Boosting; GBDT, Gradient Boosting Decision Tree; Classes: 0 indicates healthy ginseng, 1 indicates diseased ginseng.

Model optimization improves the recognition ability of the models. The discriminable ability of the 4 models before and after model optimization were listed in **Table S2**. After parameter adjustment, the accuracy of the ADA and GBDT were improved. The accuracy of Boosting’s ADA model was 88%, which was the lowest, indicating the classification effect of this model was slightly worse than the others. Compared with ADA, the accuracy of GBDT model was 4 percent higher, and the bagging strategy-based RF algorithm was 8 percent higher. ET model had the best performance with accuracy of 98%, precision of 98%, recall of 100% and F1-Score of 99%, suggesting it to be the appropriate model for classification. According to the ROC curves (**Figure 6A**), the AUC of the model of RF and ET, which were based on the bagging strategy, were relatively high, reaching 98.8% and 99.8%, respectively; and the AUC of the models of ADA and GBDT, which were based on the boosting strategy, were relatively low, reaching 84.1% and 97.0%, respectively. It was worth noting that the bagging strategy had an advantage over the boosting strategy on this dataset. According to the confusion matrices (**Figure 6B**), the overall prediction effect of all models for class 1 was better than class 0, indicating that it had a better recognition effect on ginseng root disease. The f1-score of all models in class 1 were greater than 0.9 (**Figure 6B**), meaning that all models achieved a balance between precision and recall. Among the four models, the ET model has the highest f1-score of 0.99, precision of 0.98 and recall of 1.0 (**Figure 6B**), which was taken to be the optimal model for evaluation the early detection of ginseng root diseases. Similar to the results of AUC, the models of the bagging strategy (RF and ET) were better than the models of the boosting strategy (GBDT and ADA) on this dataset.

#### 3.3.3 The determination of the range of hyperspectral bands of importance

**Figure 7:**
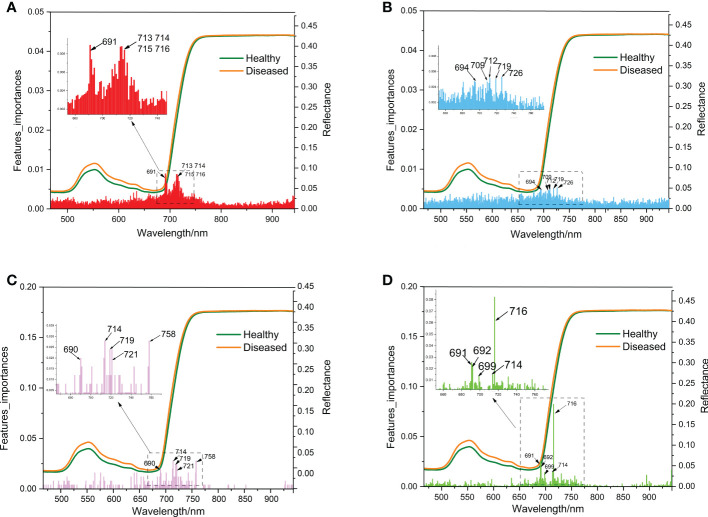
Schematic diagram of wavelength importance analysis of the 4 models (**A**: RF, **B**: ET, **C**: ADA, **D**: GBDT) same as **Figure 6**.

The hyperspectral bands of ginseng leaves measured in this experiment ranged from 325 to 1 075 nm, with a total of 750 bands. After pre-processing, 490 bands were selected as 490 variables. After processing these 490 variables by RF, ET, ADA, and GBDT algorithms, it was concluded that the most important bands for each model were 691 nm, 726 nm, 714 nm, and 716 nm, respectively (**Figure 7**). The most important bands were all ranged between 690-726 nm (**Figure 7**). Although the high randomness of the RF and ET models resulted in low band importance scores (less than 0.01), 690-726 nm was also identified within the top 5 bands of importance for these two models (**Figure 7**). As a result, in the hyperspectral reflectance of ginseng leaves, the differences of bands close to 690-726 nm were most likely to indicate whether a ginseng root was diseased or not, and this range of bands can be considered as the hyperspectral characteristic bands for ginseng root disease detection.

### 3.4 Model validation

According to the evaluation of various indicators of RF, ET, ADA and GBDT models, the ET model was selected as the early detection model for ginseng root diseases. The hyperspectral reflectance of leaves (with no symptoms) of another 12 plants growing in the nearby field were collected and were used for model validation. Among the 12 plants, 9 were detected to be diseased ones and the other 3 were considered to be healthy according to the models (**Table 2**). After harvest, these 12 ginsengs were diagnosed visually (**Figure S2**), 11 were found to be diseased and 1 was healthy (**Table 2**). The model correctly identified the 9 diseased ginsengs and the 1 healthy ginseng. Only two plants (No. 1 and 7, **Table 2**), which were actually diseased ones, were mistakenly predicted to be the healthy ones. Thus, the accuracy of this model for these 12 ginsengs is 83.3%.

**Table 2:** Root diseases prediction of 12 ginsengs in another plot by the ET model.

No. of Ginsengs	Prediction by the model	Detection after sampling	Correctness of the model
1	0	Di	No
2	1	Di	Yes
3	1	Di	Yes
4	1	Di	Yes
5	1	Di	Yes
6	1	Di	Yes
7	0	Di	No
8	1	Di	Yes
9	1	Di	Yes
10	1	Di	Yes
11	1	Di	Yes
12	0	He	Yes

Model prediction: 0 refers to the healthy root, whereas 1 refers to the diseased root. After sampling, whether the root of each ginseng was healthy (He) or diseased (Di), was identified by vision.

## 4 Discussion

The development of root disease is a gradual process. It is generally believed that symptoms on leaves appear much later until the diseases of the root are severe to a certain extent (Zhou et al., 2017). Surprisingly, our research found that when the root is seriously rotted, there are still ginsengs with leaves showing no symptoms (**Figure 2D**). Along with the visual observation, there were no significant changes in the physiological traits, like SPAD and antioxidant enzyme activities (**Figure 3**). The insignificantly changed SPAD indicated that the chlorophyll contents of leaves were not affected and even the photosynthesis system may still be well functioning when roots were infected. Even VIs including MSR, mNDVI1, VOG1, CRI1 and CRI2 were not significantly affected in these leaves, confirming the changes of leaves caused by root diseases were very slight. It is not clear how leaves could keep healthy when roots are already severely rotted, but it reminds us that the macro symptoms and even physiological traits of leaves may not reflect the status of roots and a more accurate detection method such as the one based on the spectroscopic information is necessary.

The application of hyperspectral remote sensing technology combined with ML is prospectively used in detecting diseases of an increasing number of crops (Lowe et al., 2017; Yang et al., 2020; Tian et al., 2021). So far, this technology has not been widely used in disease detection of medicinal plants. Most disease detection and identification of crops or other plants are performed on images of plant tissues where the diseases occur with visible symptoms (Singh et al., 2018; Singh et al., 2021), such as leaf blast and false smut infection of rice (An et al., 2021; Tian et al., 2021) and apple fire blight disease (Jarolmasjed et al., 2019). However, the medicinal parts of most medicinal plants, like ginsengs, are roots, whose hyperspectral reflectance data cannot be collected directly and non-constructively. Calamita et al. (2021) found significant differences in the near-infrared spectral region of leaves between healthy and root-rot grape plants by the naive bayes algorithm, which showed 90% accuracy in the identification of healthy and diseased plants, indicating the possibility for the diagnosis of root rot in plants by applying hyperspectral reflectance from leaves. However, such detection method is rarely mentioned by previous researchers and has not been reported in medicinal plants. In this study, we established a method to detect ginseng root diseases by collecting and analysing the hyperspectral reflectance data of leaves. Since the relationship between leaves and diseased root is still unclear, this indirect detection highly demands on the precision of data collection and analysis. Thus, the recognition of the region of characteristic bands and a proper method of ML processing are very important.

We found visible (460-760 nm) and near-infrared (760-950 nm) spectral reflectance of leaves played an important role in monitoring ginseng growth (**Figure 5**). Based on the data collected from leaves from this region and the visual detection of ginseng roots after harvest, ML algorithms were used to construct the model of root diseases detection. The combination of non-destructively acquired hyperspectral reflectance data and ML algorithms can recognize the tiny changes of hyperspectral reflectance of the asymptomatic patients, that greatly improves the classification accuracy of the model (Sankaran et al., 2012; Abdulridha et al., 2022). For example, the logistic regression-based ML algorithms by Appeltans et al. (2021) obtained the accuracy of automatically labelled *Phytophthora infestans* is 98.80%, wheat *Puccinia striiformis* and *Puccinia triticina* are 97.69% and 96.66%, respectively. The four ML models of RF, ET, ADA, and GBDT used in this study all belonged to EL have achieved high accuracy in the detection of ginseng root diseases (> 85%). EL is a commonly used ML algorithm in processing hyperspectral datasets (Wei et al., 2020; Ekramirad et al., 2022). Its advantage is to organize several simple algorithms to jointly determine the final performance. Among the models, RF and ET are based on bagging strategy and mainly optimize the robustness (variance) of the model (Breiman, 2001; Svetnik et al., 2003). For example, RF hyperspectral model can better predict heavy metal distribution in agricultural soil (Tan et al., 2020). Whereas ADA and GBDT are based on boosting strategy and mainly optimize the precision (bias) of the model (Freund and Schapire, 1997). For example, the GBDT model can validly classify apple bruising times (Pan et al., 2019). In this study, after parameter adjustment, the models of the bagging strategy (RF and ET) were more suitable for this dataset. The accuracy of ET model was highest of 98% and the AUC of ET model is as high as 99.8% (**Figure 6**). This is consistent with the good generalization ability of the ET algorithm, that successfully classified seven types of Spanish honeys with single botanical origins (Mateo et al., 2021).

Besides model selection, the pre-processing method is also strongly affected the model accuracy. The partial least squares discriminant analysis achieves the best results in SNV-processed *Paris yunnanensis* data of different origins (Pei et al., 2018). For the ET model in this study, the accuracy of the SG pre-processing method is 19.52 percentage higher than that of the 2D pre-processing method (**Table 1**). Based on the contribution rate of the models, the most important bands collected from ginseng leaves were all concentrated in the range of 690-726 nm (**Figure 7**). Combined with the analysis of hyperspectral VIs (**Figure 4**), the significant differences of NDVI were also associated with this range. The reflectance at the red edge position is suggested to be used to evaluate the structural changes and physiological degradation of leaves (Vescovo et al., 2012; Liu et al., 2014). Thus, the changes in the position of red edges in ginseng leaves may be the results of unobservable changes in leaf structure caused by lesions of ginseng root. Therefore, the range of characteristic bands for early detection of ginseng root diseases was further narrowed.

**Figure 8:**
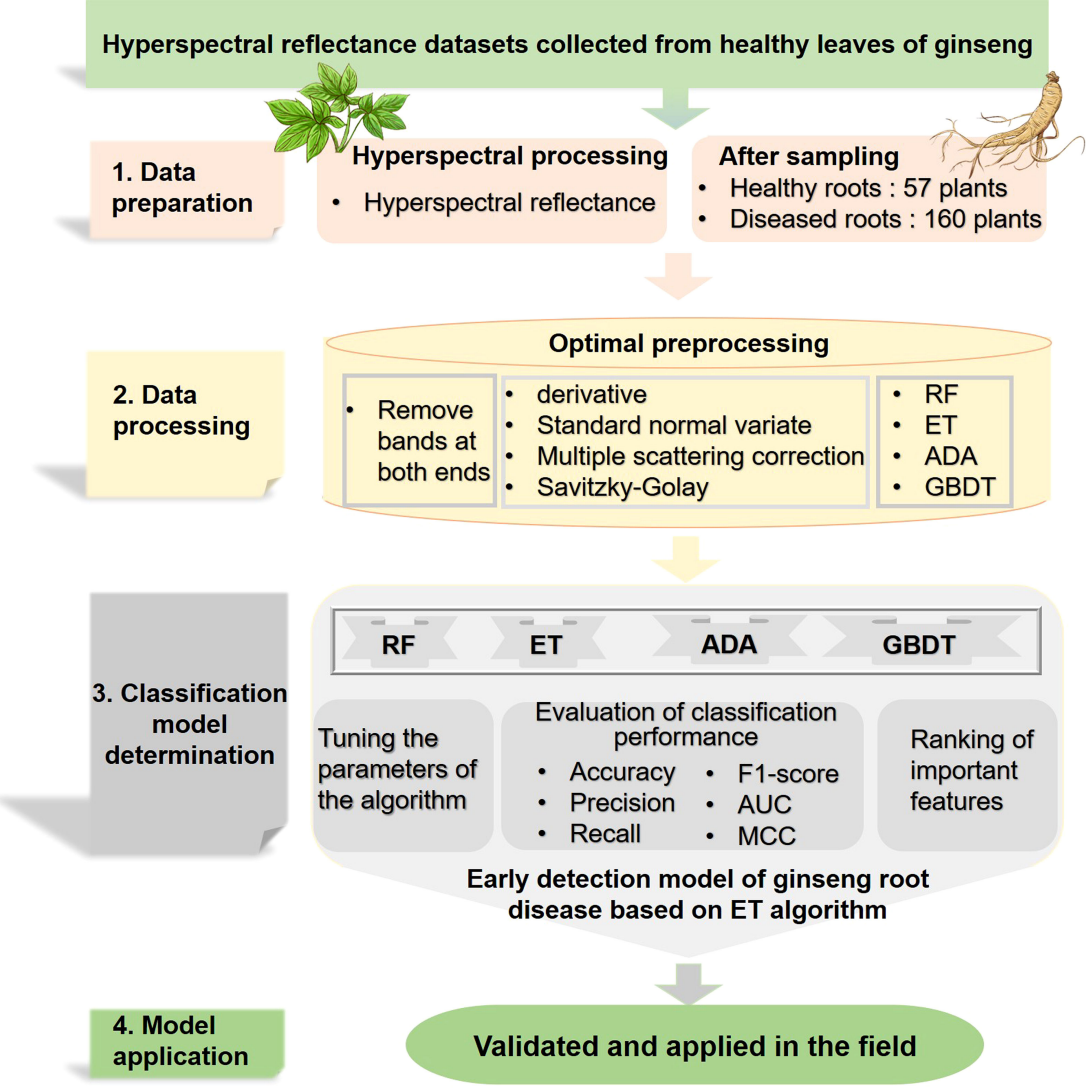
Schematic diagram of the classification method based on the ET algorithm for spectral detection of ginseng root diseases. same as **Figure 6**; AUC, area under the curve; MCC, Matthew’s correlation coefficient.

This study explored the detection method of root diseases based on the hyperspectral reflectance of asymptomatic ginseng leaves. The method was built based on the combination of non-destructively acquired hyperspectral data from healthy leaves and the ML classification algorithms. After pre-processing and optimizing the data set, classification models combined with the indicators were evaluated. Finally, the method for early detection of ginseng root disease was built and validated. The pipeline of the construction of the method was shown in **Figure 8**. Besides, ginsengs from another plot with healthy leaves were also collected and were used to validate the model. Though with a limited number of samples, the model achieves a correct rate of 83.3%, showing the effectiveness of the method. Upon the high detection rate of the method, the method can still be improved in many aspects. The hyperspectral reflectance can be affected by many factors, such as the ages of ginseng, the development stages of ginsengs, the types or causes of diseases, the severity of diseases as well as the changes of environments. Thus, based on the effectiveness of the simple model we built in this study, it is promising to develop an integrated algorithm based on data collected during the precise identification phase of root disease development. In addition to the two groups of healthy and diseased, more variables such as the development stages of ginsengs, the environmental factors, the type of the diseases and the severity of the diseases can be brought into the model. To be more precisely, hyperspectral data can be collected in the manual interventions conditions, such as different stages of ginsengs being pathogenic inoculated or ginsengs growing under certain abiotic stresses, that lead to a certain type of ginseng root diseases. Thus, a lot more data are expected to be collected, and a hyperspectral reflectance database of ginseng root diseases database can be built. Since the training of ML models requires large amount of data (Tsaftaris et al., 2016), comprehensive algorithms are supposed to be developed and improved based on the database. In addition, the development of the detection method could be combined with pathological studies, which would provide basic knowledge of the mechanism how hyperspectral reflectance are related to the diseased roots, aiding the construction of ML models. To summarize, based on the detection method of ginseng root diseases constructed by the hyperspectral reflectance of asymptomatic ginseng leaves that has been proposed in this study, a comprehensive hyperspectral reflectance database is expected to be built in the future together with the development of ML models, in order to accurately identify the type, onset time, severity of underground diseases of ginsengs and to perform timely treatments to reduce production loss. Early detection of root diseases is essential as timely excavation of diseased ginseng in the early stages of the disease can prevent further infection of other plants. This will help to provide new methods for disease detection in other root plants and also provide ideas for the development of more portable and simple field detection equipment. Equipping hyperspectral remote sensing equipment with intelligent robots for self-service detection will also be a future goal for the development of smart agriculture in the field. By establishing abiotic stress tests, combined with hyperspectral remote sensing technology to further explore the optimal cultivation conditions for ginseng root growth, the environmental conditions for ginseng root disease development will be blocked at source and root disease prevention will be achieved. In the field cultivation of ginseng, the diseased ginseng can be harvested according to the predicted results based on our established model for early detection of root diseases, further completing the identification of ginseng root disease pathogens, exploring the pathogenic mechanisms of fungi and bacteria, screening for efficient and non-polluting antibacterial substances, and finally achieving the prevention and control of the disease.

## Data availability statement

The datasets presented in this study can be found in online repositories. The names of the repository/repositories and accession number(s) can be found below: https://github.com/yang1rq/ginseng/tree/master.

## Author contributions

XL, DaY, and GZ conceived, designed, processed, analysed, and interpreted the experiments. GZ, DoY, and JW acquired the data. GZ, YP and RY analysed the data and prepared the manuscript. GZ, LX, ZF, YW and DG edited the manuscript. All authors contributed to the article and approved the submitted version.

## Funding

The research was funded by National Key Research and Development Program of China (No. 2019YFC1710601), Scientific and technological innovation project of China Academy of Chinese Medical Sciences (CI2021A04505), National Natural Science Foundation of China (No. 81891013) and the Fundamental Research Funds for the Central public welfare research institutes (ZZ15-YQ-031, ZXKT20133).

## Conflict of interest

The authors declare that the research was conducted in the absence of any commercial or financial relationships that could be construed as a potential conflict of interest.

## Publisher’s note

All claims expressed in this article are solely those of the authors and do not necessarily represent those of their affiliated organizations, or those of the publisher, the editors and the reviewers. Any product that may be evaluated in this article, or claim that may be made by its manufacturer, is not guaranteed or endorsed by the publisher.
