# Supplementary material for: A non-destructive testing method for early detection of ginseng root diseases using machine learning technologies based on leaf hyperspectral reflectance
Source: Front Plant Sci. 2022 Nov 17;13:1031030. doi: 10.3389/fpls.2022.1031030 (PMC9714554; doi:10.3389/fpls.2022.1031030)
Supplement: Supplementary file 1 [file DataSheet_1.docx]

Supplementary Material

# Supplementary Figures and Tables

**
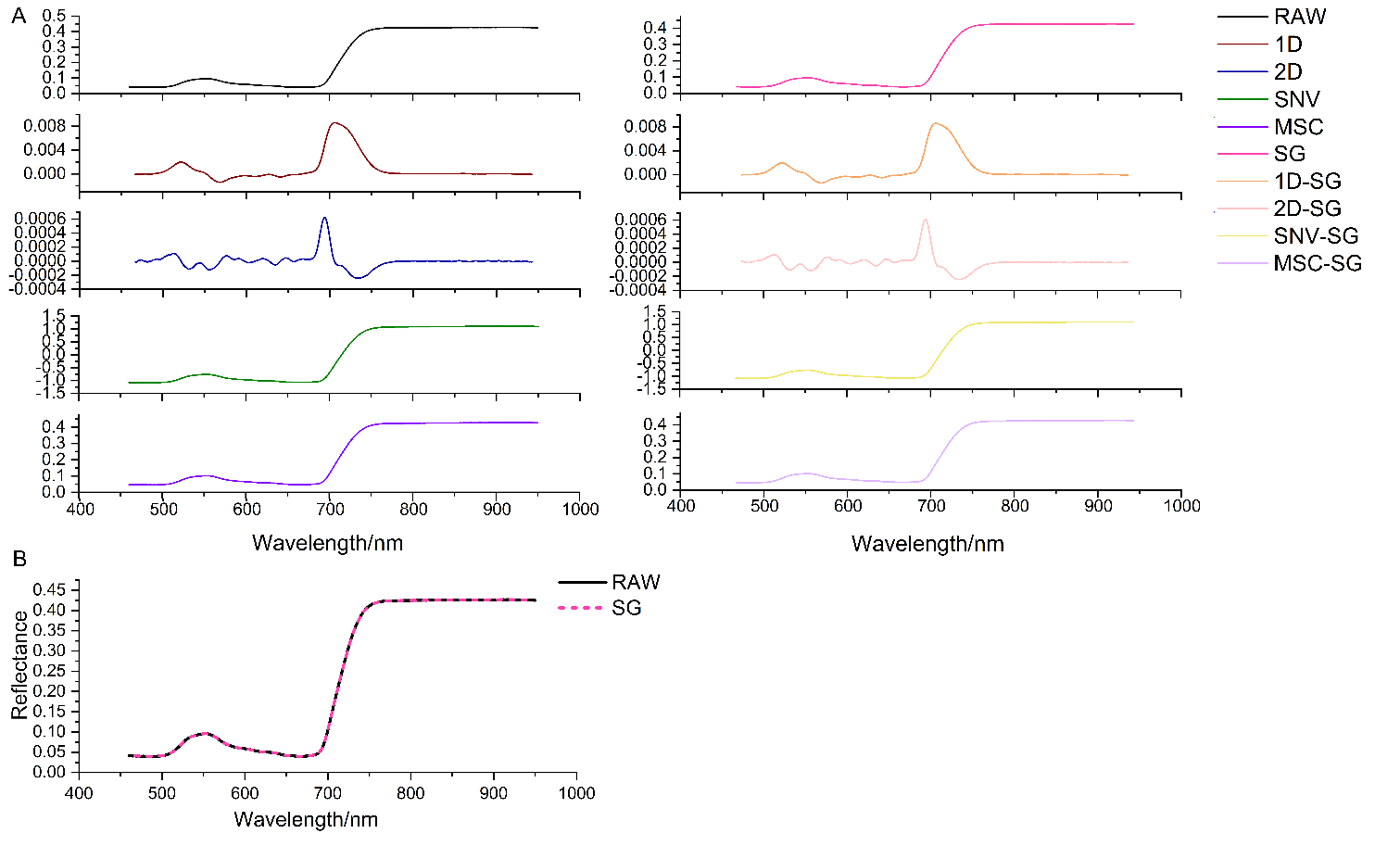
**

**Supplementary Figure 1.** Changes of hyperspectral reflectance with different preprocessing methods

A is the result of 10 preprocessing methods, B is the result of hyperspectral reflectance after SG Abbreviations: RF, Random forest; ET, Extremely randomized trees; ADA, Adaptive boosting; GBDT, Gradient boosting decision tree; RAW, Raw dataset; 1D, first derivative; 2D, Second derivative; SNV, Standard normal variate; MSC, Multiple scattering correction; SG, Savitzky golay; 1D-SG, first derivative- savitzky golay; 2D-SG, Second derivative – savitzky golay; SNV-SG, Standard normal variate – savitzky golay; MSC-SG, Multiple scattering correction- savitzky golay

**
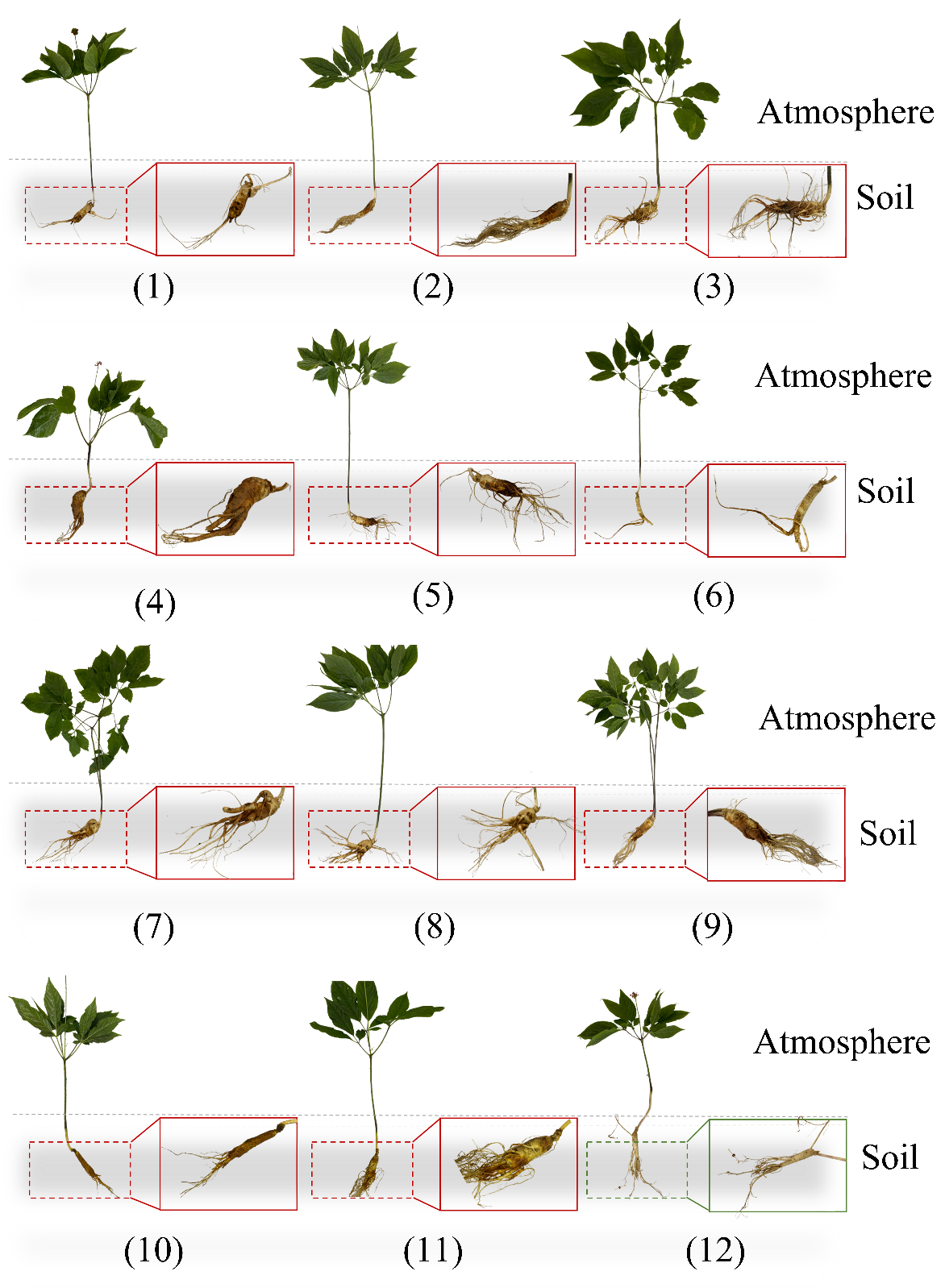
**

**Supplementary Figure 2.** Photos of the aboveground and underground parts of 12 ginseng plants used for independently verification

**Supplementary Table1.** Vegetation indices used for measuring reflectance changes between leaves from healthy and diseased ginsengs

| **Vegetation Index** | **Abbreviation** | **Equation** | **Related to** |
| --- | --- | --- | --- |
| Red Edge Normalized Difference Vegetation Index | NDVI | (R_750_-R_705_)/(R_750_+R_705_) | Vegetation |
| Modified Red Edge Simple Ratio Index | MSR | (R_750_-R_445_)/(R_705_+R_445_) | Vegetation |
| Modified Red Edge Normalized Difference Vegetation Index | mNDVI | (R_750_-R_705_)/2(R_705_-R_445_) | Vegetation |
| Vogelmann Red Edge Index1 | VOG1 | R_740_/R_720_ | Vegetation |
| Carotenoid Reflectance Index 1 | CRI1 | R_510_^-1^-R_550_^-1^ | Carotenoid |
| Carotenoid Reflectance Index 2 | CRI2 | R_510_^-1^-R_700_^-1^ | Carotenoid |
| Anthocyanin Reflectance Index 1 | ARI1 | R_550_^-1^-R_700_^-1^ | Anthocyanins |
| Anthocyanin Reflectance Index 2 | ARI2 | R_550_^-1^-R_700_^-1^ | Anthocyanins |

**Supplementary Table2.** Discrimination results of the four models before and after parameter adjustment

| Evaluation indicators | Before parameter adjustment | | | | After parameter adjustment | | | |
| --- | --- | --- | --- | --- | --- | --- | --- | --- |
|  | RF | ET | ADA | GBDT | RF | ET | ADA | GBDT |
| Accuracy | 0.96 | 0.98 | 0.79 | 0.87 | 0.96 | 0.98 | 0.88 | 0.92 |
| Precision | 0.96 | 0.97 | 0.81 | 0.87 | 0.96 | 0.98 | 0.89 | 0.91 |
| Recall | 0.98 | 1.00 | 0.94 | 0.97 | 0.98 | 1.00 | 0.95 | 0.98 |
| F1-Score | 0.97 | 0.99 | 0.87 | 0.92 | 0.97 | 0.99 | 0.92 | 0.95 |
| AUC | 0.98 | 1.00 | 0.84 | 0.95 | 0.99 | 1.00 | 0.93 | 0.97 |
| MCC | 0.89 | 0.95 | 0.44 | 0.67 | 0.90 | 0.96 | 0.69 | 0.79 |

RF: Random Forest; ET: Extremely randomized trees; ADA: Adaptive Boosting; GBDT: Gradient Boosting Decision Tree; AUC: area under the curve; MCC: Matthew’s correlation coefficient
